# Rat hair-follicle-associated pluripotent (HAP) stem cells can differentiate into atrial or ventricular cardiomyocytes in culture controlled by specific supplementation

**DOI:** 10.1371/journal.pone.0297443

**Published:** 2024-01-26

**Authors:** Nanako Takaoka, Michiko Yamane, Ayami Hasegawa, Koya Obara, Kyoumi Shirai, Ryoichi Aki, Hiroyasu Hatakeyama, Yuko Hamada, Nobuko Arakawa, Manabu Tanaka, Robert M. Hoffman, Yasuyuki Amoh

**Affiliations:** 1 Department of Dermatology, Kitasato University Graduate School of Medical Sciences, Kitasato University School of Medicine, Minami Ward, Sagamihara, Japan; 2 Department of Dermatology, Kitasato University School of Medicine, Kitasato University School of Medicine, Minami Ward, Sagamihara, Japan; 3 Department of Physiology, Kitasato University School of Medicine, Kitasato University School of Medicine, Minami Ward, Sagamihara, Japan; 4 Bio-Imaging Center, Kitasato University School of Medicine, Minami Ward, Sagamihara, Japan; 5 AntiCancer, Inc., San Diego, CA, United States of America; 6 Department of Surgery, University of California, San Diego, CA, United States of America; Cinvestav-IPN, MEXICO

## Abstract

There has been only limited success to differentiate adult stem cells into cardiomyocyte subtypes. In the present study, we have successfully induced beating atrial and ventricular cardiomyocytes from rat hair-follicle-associated pluripotent (HAP) stem cells, which are adult stem cells located in the bulge area. HAP stem cells differentiated into atrial cardiomyocytes in culture with the combination of isoproterenol, activin A, bone morphogenetic protein 4 (BMP4), basic fibroblast growth factor (bFGF), and cyclosporine A (CSA). HAP stem cells differentiated into ventricular cardiomyocytes in culture with the combination of activin A, BMP4, bFGF, inhibitor of Wnt production-4 (IWP4), and vascular endothelial growth factor (VEGF). Differentiated atrial cardiomyocytes were specifically stained for anti-myosin light chain 2a (MLC2a) antibody. Ventricular cardiomyocytes were specially stained for anti-myosin light chain 2v (MLC2v) antibody. Quantitative Polymerase Chain Reaction (qPCR) showed significant expression of *MLC2a* in atrial cardiomyocytes and *MLC2v* in ventricular cardiomyocytes. Both differentiated atrial and ventricular cardiomyocytes showed characteristic waveforms in Ca^2+^ imaging. Differentiated atrial and ventricular cardiomyocytes formed long myocardial fibers and beat as a functional syncytium, having a structure similar to adult cardiomyocytes. The present results demonstrated that it is possible to induce cardiomyocyte subtypes, atrial and ventricular cardiomyocytes, from HAP stem cells.

## Introduction

We discovered hair-follicle-associated pluripotent (HAP) stem cells twenty years ago [1]. HAP stem cells are located in the hair follicle bulge area and have been differentiate into neurons, glia, keratinocytes, adipocytes, smooth muscle cells, melanocytes, dopaminergic neurons, and beating cardiomyocytes [2–11]. We succeeded in differentiating mature beating cardiomyocyte sheets with long myocardial fibers from mouse and rat HAP stem cells [12, 13].

Cardiac disease is one of the leading causes of death in the world, but the mature heart muscle cannot regenerate, there are few effective treatments, and severe cases are still relying on heart transplantation. However, the number of heart transplants is not sufficient, and many patients on the waiting list for heart transplantation do not receive effective treatment. Therefore, there are urgent needs for new therapeutic agents and regenerative medicine.

Differentiation of cardiomyocytes from various stem cells has studied. Embryonic stem cells (ESC) and induced pluripotent stem cells (IPSC) have induced more differentiated cardiomyocyte subtypes (atrial and ventricular cardiomyocytes) [14–16], but there have not been sufficient studies on differentiating adult stem cell-derived cardiomyocyte subtypes.

From these studies, we considered that it was possible to induce more differentiated cardiomyocyte subtypes (atrial and ventricular cardiomyocytes) from HAP stem cells, which are adult stem cells. Based on previous studies, we cultured HAP stem cells by adding CSA to improve the differentiation efficiency of cardiomyocytes and cardiomyocyte progenitor cells, Wnt to affect the differentiation of the left and right chambers of the heart, and VEGF to promote angiogenesis and vessel formation. Immunostaining and Quantitative Polymerase Chain Reaction (qPCR) showed that differentiated cardiomyocytes expressed cardiac specific gene and proteins for atrial (*MLC2a*) and ventricular (*MLC2v*) cardiomyocytes, demonstrated differentiation of atrial and ventricular cardiomyocytes.

In the present study, HAP stem cells were cultured with specific supplements to selectively differentiate them into atrial or ventricular cardiomyocytes with long myocardial fibers without genetic manipulation.

HAP stem cells are readily available adult stem cells and the present study demonstrating their future clinical potential for cardiac regeneration.

## Material and methods

### F344/jcl rats

A total of 18 four-week-old F344/jcl female healthy rats (weight over 50g) (CLEA Japan, Tokyo Japan) were used for the vibrissa hair-follicle isolation. The rats were maintained as 24 ± 1°C, relative humidity of 50–60%, and 14 hours of light 10 hours of dark intervals. Euthanasia was performed by CO_2_ asphyxiation followed by cervical dislocation according to the American Veterinary Medical Association (AVMA) Guidelines. All procedures involving the rats were in accordance with the Guidelines of the United States National Institutes of Health (NIH) and with the Animal Research: Reporting of *In Vivo* Experiments (ARRIVE) guidelines and were approved by the Animal Experimentation and Ethics Committee of Kitasato University School of Medicine (Approval No. 2022–043). Every effort was made to minimize animal suffering and reduce the number of animals used.

### Isolation of rat vibrissa hair follicles

Processing of rat vibrissa hair follicles was performed as previously reported [13]: F344/jcl rats were anesthetized with a combination of 0.375 mg/kg medetomidine, 2.0 mg/kg midazolam and 2.5 mg/kg butorphanol. The upper lip containing the vibrissa hair follicle was excised and placed in a petri dish with DMEM (#D6429, Sigma-Aldrich, St. Louis, MO, USA) containing 10% fetal bovine serum (FBS), 50 μg/ml gentamicin (#15750–060, GIBCO, Grand Island, NY, USA), 2 mM L-glutamine (#25030, GIBCO), 10 mM HEPES (#H0887, Sigma-Aldrich). The inner surface of the excised upper lip was exposed, and under a binocular microscope, all vibrissa hair follicles were carefully pulled out from the pad one by one, with a fine forceps. Approximately 20 hair follicles were collected from each pad. The tissues surrounding the separated hair follicles were carefully removed, the hair follicles were divided into three equal parts, and only the upper part, containing the bulge area, was placed in culture as described below.

### Atrial or ventricular cardiomyocytes differentiation from rat HAP stem cells

**Fig 1:**
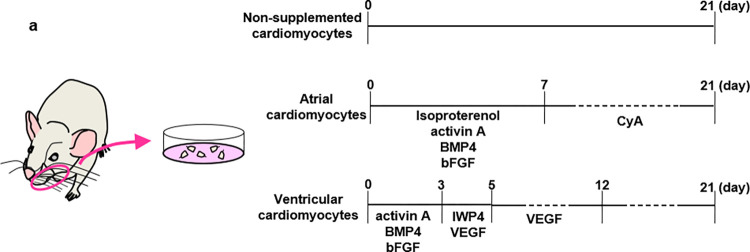
Shema for rat HAP stem cells differentiation into atrial or ventricular cardiomyocytes. (a) The upper parts of rat vibrissa hair follicles were cultured and supplemented as described.

Induction of differentiation of HAP stem cells into cardiomyocytes was performed as previously reported [9]: The upper parts of the hair follicles were placed with Matrigel^Ⓡ^ (#356231, Corning Incorporated, Corning, NY, USA) culture in 35 mm glass-bottom collagen-coated culture dishes (#P35GCOL-0-10-C, MatTeK corporation, Ashland, MA), in DMEM containing 10% FBS, 50 μg/ml gentamycin, 2 mM L-glutamine and 10 mM HEPES. Atrial cardiomyocytes were induced by adding isoproterenol (3 μM) (#I6504, Sigma-Aldrich), activin A (10 ng /ml) (#338-AC-010, HumanZyme, Chicago, IL, USA), bone morphogenetic protein 4 (BMP4) (10 ng /ml) (#120-05ET, HumanZyme), and basic fibroblast growth factor (bFGF) (5 ng /ml) (#GF003, Millipore, Temecula, CA, USA) for 7 days, followed by cyclosporin A (CSA) (1 μg/mL) (#AG-CN2-0079, AdipoGen Life Sciences, San Diego, CA, USA) for 14 days in 10% FBS-DMEM. Ventricular cardiomyocytes were induced by adding activin A (12 ng /ml), BMP4 (5 ng /ml), and bFGF (5 ng /ml) for 3 days, followed by inhibitor of Wnt production-4 (IWP4) (1 μM) (#13954,Cayman,ANN ARBOR,MI, USA) and vascular endothelial growth factor (VEGF) (10 ng /ml) (#100–20, PEPROTECH, Cranbury, NJ, USA) for 4 days, then VEGF (5 ng /ml) alone for 6 days in StemPro-34 (#10639011, GIBCO) containing 50 μg/ml gentamicin, 2 mM L-glutamine, transferrin (150 μg/ml) (#T0665, Sigma-Aldrich), ascorbic acid (50 μg/ml) (#49752, Sigma-Aldrich), and 2-mercaptoethanol (0.1mM) (#21985–023, gibco). Supplements were added every 3 days and all cultures were grown for 21 days (Fig 1A). Cardiomyocytes were visualized and recorded with a microscope video camera (VCE-i700T, Shodensha, Osaka, Japan), and videos were edited with Microsoft Clipchamp (Microsoft Corporation, WA, USA).

### Immunofluorescence staining and live-cell imaging

Immunofluorescence staining was performed as previously reported [13]: Primary antibodies used were: anti-cardiac troponin I (cTnI) rabbit polyclonal antibody (1:200, #PAA478Ra01, Cloud-Clone Corp, Houston, TX, USA); anti-cardiac troponin T (cTnT) mouse monoclonal antibody (1;500, #GTX28295, GeneTex, Irvin, CA, USA) to prove cardiomyocytes; anti-myosin light chain 2a (MLC2a) mouse monoclonal antibody (1:40, #565496, BD Biosciences, San Jose, CA, USA) to prove atrial cardiomyocytes; anti-myosin light chain 2v (MLC2v) rabbit monoclonal recombinant antibody (1:200, # 310 118, Synaptic Syatems, Göttingen, Germany) to prove ventricular cardiomyocytes. Secondary antibodies used were: goat anti-rabbit IgG conjugated with Alexa Flour 568^®^ (1:400, #,A11011, Molecular Probes, Eugene, Oregon, USA); goat anti-mouse IgG conjugated with Alexa Fluor 488^®^ (1:400, #A-11001, Molecular Probes); goat anti-mouse IgG conjugated with Alexa Flour 568^®^ (1:400, #A11004, Molecular Probes); goat anti-rabbit IgG conjugated with Alexa Flour 488^®^ (1:400, #A11008, Molecular Probes). 4’, 6-diamino-2-phenylindole, dihydrochloride (DAPI) (#SE196, DOJINDO, Kumamoto, Japan) was used for counter staining. Images of stained cells were obtained with an LSM 710 microscope (Carl Zeiss, Oberkochen, Germany). The images were analyzed by LSM software ZEN (Carl Zeiss). For cell counting, image processing was with image J software (version 1.53f51: National Institutes of Health, Bethesda, Maryland, USA) [17]. Live-cell imaging was performed with SiR-actin Kit (#CY-SC1, Cytoskeleton, DENVER, CO, USA). Added 1 μM SiR-actin to the culture medium and placed in incubator at 37°C for 1 hour. Imaging experiments were performed with an inverted microscope (IX83, Evident, Tokyo, Japan) equipped with an EMCCD camera (iXon ultra, Andor, Belfast, UK), a spinning disk confocal unit (CSU-X1, Yokogawa, Japan), a z-drift compensator (IX3-ZDC2, Evident), and an oil-immersion objective lens (UPLFLN40xO, NA1.3, Evident). Excitation was at 640 nm with a laser diode illuminator (LDI-7, Chroma, Bellows Falls, VT, USA) and the fluorescence of SiR-actin was acquired at 75 frames/second with iQ software (Andor). Videos were edited with Microsoft Clipchamp.

### Quantitative Polymerase Chain Reaction (qPCR)

Total RNA was isolated from 21day cardiomyocytes using the RNeasy Plus Mini Kit (#74134, Qiagen, Hilden, Germany). cDNA was synthesized with QuantiTect^®^ Reverse Transcription (#205311, Qiagen) according to the manufacturer’s instructions. Quantitative PCR was performed using the Power SYBR® Green PCR Master mix (#4367659, Applied Biosystems, Waltham, MA, USA). Amplification was performed at 95°C for 10 min, followed by 40 cycles at 95°C 10 s and at 57°C for 60 s on a CFX96 Real-Time PCR Detection System (Bio-Rad, Hercules, CA, USA) and analyzed by the Delta Delta Ct method. GAPDH was used to normalize gene expressions. Forward and reverse primer sequences are shown in Table 1.

**Table 1:** Primers for qPCR.

Gene	Forward Primer	Reverse Primer
*MLC2a*	AGG CCA TCC TGA GTG CTT TC	GTC AGG TCC ATG GGT GTC AG
*MLC2v*	CGC TGA AGG CCG ACT ATG T	TCG ATC TCT TCT TTG GAG AAC CTC
*GAPDH*	GCA TCT TCT TGT GCA GTG CC	GAT GGT GAT GGG TTT CCC GT

### Transmission electron microscopy

Differentiated cells from HAP stem cells were pre-fixed with 2.5% glutaraldehyde in 0.1M sodium cacodylate buffer for 30 min at 4°C, post-fixed with 1% osmium tetroxide for 1 hour at 4°C. A biological specimen was immersed in a solution of 2% uranyl acetate for 15 min an aqueous solution after post-fixation, for en-bloc staining. The specimens were then dehydrated through graded ethanol and embedded in epoxy resin. Ultrathin sections (80 nm), double-stained with 2% uranyl acetate and Reynold’s lead citrate, were examined under electron microscopy H-7650 (Hitachi, Tokyo, Japan).

### Intracellular calcium imaging

Intracellular calcium imaging was performed as previously reported [13]: The cells were washed with Earle’s balanced salt solution (EBSS, GIBCO) containing 2 mmol CaCl_2_ at 37°C and were loaded with the calcium-sensitive dye Fluo 4-AM (1 μM) (#F312, DOJINDO, Kumamoto, Japan) and AM ester-dissolving reagent Pluronic F-127 (0.04%) (#59004, FUJIFILM Wako, Osaka, Japan) at 37°C for 20 min. Fluo 4-AM fluorescence (excitation at 495 nm and emission at 518 nm) of beating cardiomyocytes was measured every 5 msec with an LSM 710 microscope (Carl Zeiss). Images were analyzed with LSM software ZEN Time series (Carl Zeiss). The waveforms were created with Microsoft Excel for Windows version 2103.

### Statistical analysis

Statistical analysis was performed with Microsoft Excel for Windows version 2103 and JMP Pro version 16 (SAS, Cary, NC, USA). The experimental data are expressed as the mean ± SD. Statistical analysis was performed using a two-sided Student’s t-test, a two-sided Welch’s t-test, and a two-sided Mann-Whitney U test. *p* < 0.05 is considered statistically significant.

## Results

### Rat HAP stem cells differentiate into atrial or ventricular cardiomyocytes controlled by specific supplementation

**Fig 2:**
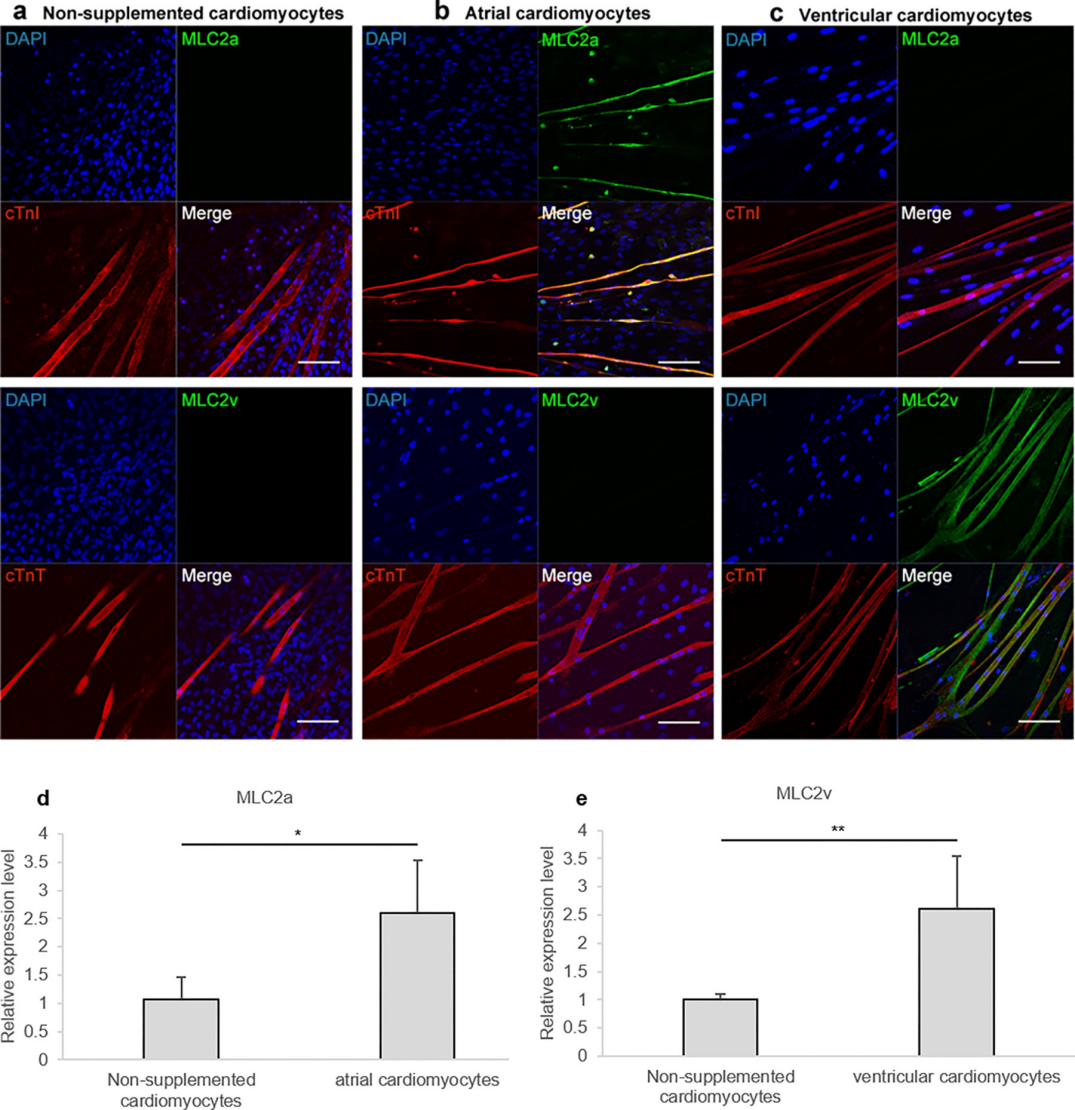
Rat HAP stem cells differentiated into atrial or ventricular cardiomyocytes. (a-c) Immunofluorescence staining of the upper parts of rat vibrissa hair follicles, which were cultured for 21 days, differentiated into cTnI (a-upper; red) and cTnT (a-lower; red)-positive non-supplemented cardiomyocytes; cTnI (b-upper; red), cTnT (b-lower; red)- and MLC2a (b; green)-positive atrial cardiomyocytes; cTnI (c-upper; red), cTnT (c-lower; red)- and MLC2v (c; green)-positive ventricular cardiomyocytes. Nuclear staining with DAPI (blue). Scale bars; 100 μm. (d, e) qPCR analyses of cardiomyocytes differentiated from HAP stem cells. n = 4 per group for non-supplemented, atrial, and ventricular cardiomyocytes. Data are presented as mean ± SD. * P < 0.05, ** P < 0.005, two-sided Student’s t-test.

Four or five the upper parts of rat vibrissa hair follicles were placed with Matrigel^Ⓡ^, cultured for 21 days with specific supplements. Immunostaining showed that non-supplemented medium resulted in rat HAP stem cells differentiating into cardiomyocytes, which stained positively with anti- cardiac-troponin-I (cTnI) (cTnI / DAPI = 17.9 ± 3.4%) and—cardiac-troponin-T (cTnT) antibody but stained negatively with anti- myosin light chain 2a (MLC2a) and—myosin light chain 2v (MLC2v) antibody (Fig 2A). In contrast, rat HAP stem cells differentiated into cTnI -and MLC2a -positive atrial cardiomyocytes, when supplemented with the combination of isoproterenol, activin A, bone morphogenetic protein 4 (BMP4), basic fibroblast growth factor (bFGF), and cyclosporine A (CSA) (cTnI / DAPI = 23.4 ± 8.9%, MLC2a / cTnI = 96.6 ± 1.4%) (Fig 2B). Rat HAP stem cells differentiated into cTnT -and MLC2v -positive ventricular cardiomyocytes, when supplemented with the combination of activin A, BMP4, bFGF, inhibitor of Wnt production-4 (IWP4), and vascular endothelial growth factor (VEGF) (cTnT/ DAPI = 6.6 ± 1.4%, MLC2v / cTnT = 83.3 ± 11.3%) (Fig 2C).

### Specific gene expression in atrial or ventricular cardiomyocytes differentiated from rat HAP stem cells

Quantitative polymerase chain reaction (qPCR) analyses showed the expression levels of *MLC2a* relative to GAPDH were 1.1 ± 0.4 for cardiomyocytes differentiated from HAP stem cells in non-supplemented medium; 2.6 ± 0.9 for atrial cardiomyocytes differentiated from HAP stem cells in culture supplemented with the combination of isoproterenol, activin A, BMP4, bFGF, and CSA. The expression levels of *MLC2v* relative to *GAPDH* were 1.0 ± 0.1 for cardiomyocytes differentiated from HAP stem cells in non-supplemented medium; 2.6 ± 0.9 for ventricular cardiomyocytes differentiated from HAP stem cells in culture supplemented with the combination of activin A, BMP4, bFGF, IWP4, and VEGF. The expression levels of *MLC2a* relative to *GAPDH* were significantly increased in atrial cardiomyocytes compared to non-supplemented cardiomyocytes (Fig 2D) (*p* = 0.02). The expression levels of *MLC2v* relative to *GAPDH* were significantly increased in ventricular cardiomyocytes compared to non-supplemented cardiomyocytes (Fig 2E) (*p* = 0.002).

### Structural maturation of atrial or ventricular cardiomyocytes differentiated from rat HAP stem cells

**Fig 3:**
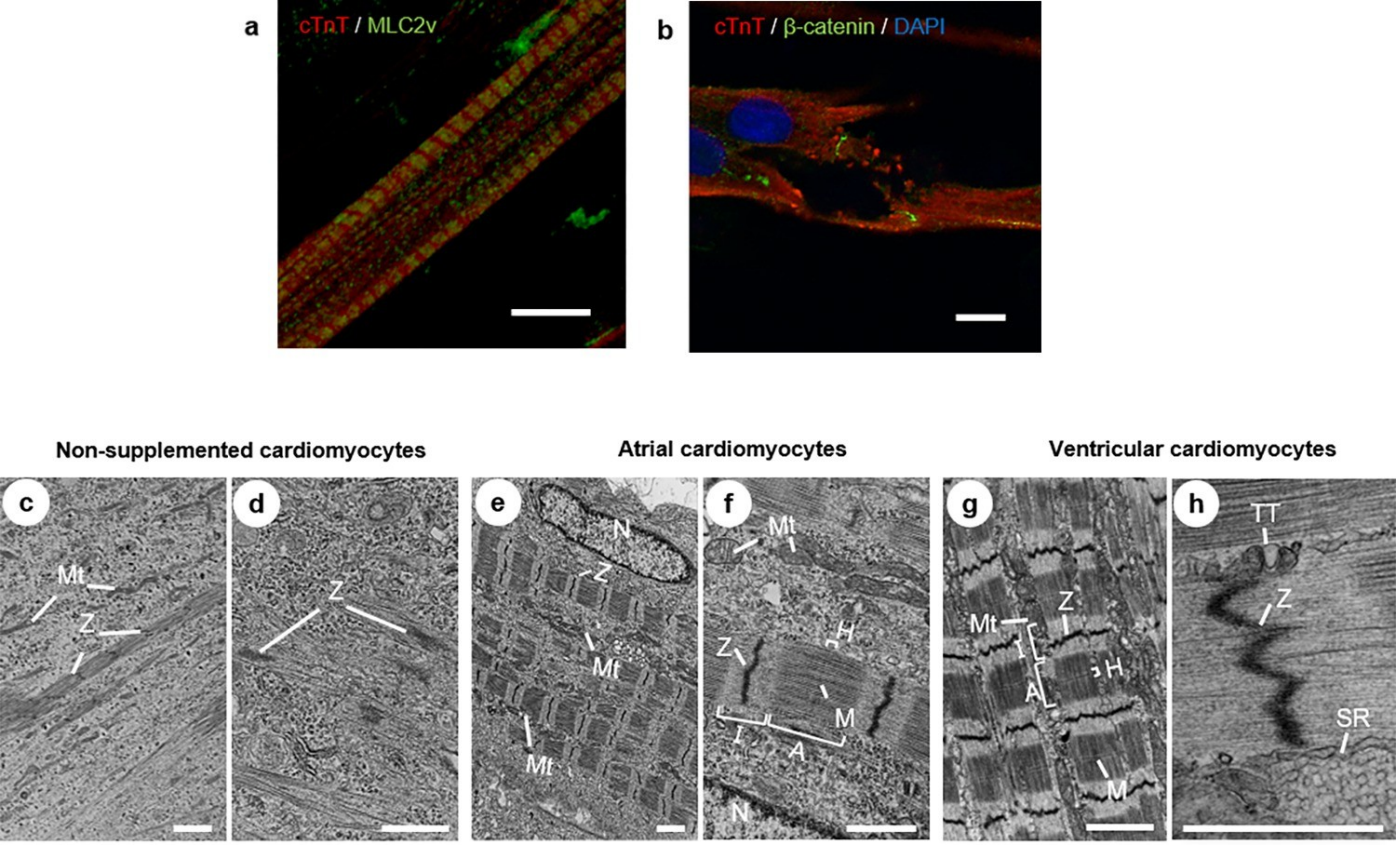
Atrial and ventricular cardiomyocytes differentiated from rat HAP stem cells had structures of mature cardiomyocytes. (a) Double immunostaining of cTnT (red)- and MLC2v (green)-positive images, cTnT in the I-band and MLC2v in the A-band showed an alternating pattern of sarcomeres. Scale bars; 10 μm. (b) Double immunostaining of cTnT (red)- and β-catenin (green)-positive images of ventricular cardiomyocytes differentiated from HAP stem cells. Nuclear staining with DAPI (blue). Scale bars; 10 μm. (c-h) Transmission electron microscopy images of cardiomyocytes differentiated from HAP stem cells. (c, d) Non-supplemented cardiomyocytes. Microfibrils with scattered Z-bands (Z) and small-sized mitochondria (Mt). (e-h) Atrial cardiomyocyte (e, f) and ventricular cardiomyocyte (g, h) differentiated from HAP stem cells. Microfibrils with regularly Z-bands (Z). A-bands (A), I-bands (I), H-bands (H) and M-bands (M) were also organized. Large mitochondria (Mt) with complex internal structures. Ventricular cardiomyocytes had T-tubule-like structures (TT) and sarcoplasmic reticulum (SR). N: nuclear. (c, e, g) Scale bars; 2 μm. (d, f, h) Scale bars; 1 μm.

Atrial or ventricular cardiomyocytes differentiated from HAP stem cells had sarcomere structures consisting of actin and myosin filaments (Fig 3A). In addition, ventricular cardiomyocytes differentiated from HAP stem cells showed intervening plates, which stained positively with β-catenin (Fig 3B). Transmission electron microscopy showed that non-supplemented cardiomyocytes differentiated from HAP stem cells contained myofibrils, but there was no clear alignment and irregular structure with scattered Z-bands, and no T-tubule structure (Fig 3C and 3D). Atrial or ventricular cardiomyocytes differentiated from HAP stem cells had sarcomere structures, with regularly arranged Z-bands and organized A-and I-bands, as well as H-and M-bands (Fig 3E–3G). Mitochondria were located alongside the sarcomeres. Ventricular cardiomyocytes differentiated from HAP stem cells also had T-tubule-like structures identified along the Z-bands (Fig 3H). SiR-actin live-cell imaging also showed that non-supplemented cardiomyocytes differentiated from HAP stem cells were beating with unclear actin skeletons. On the other hand, atrial and ventricular cardiomyocytes were beating with a very clear actin skeleton (S1 Video).

### Characteristics of Ca^2+^-dependent beating of atrial and ventricular cardiomyocytes differentiated from rat HAP stem cells

**Fig 4:**
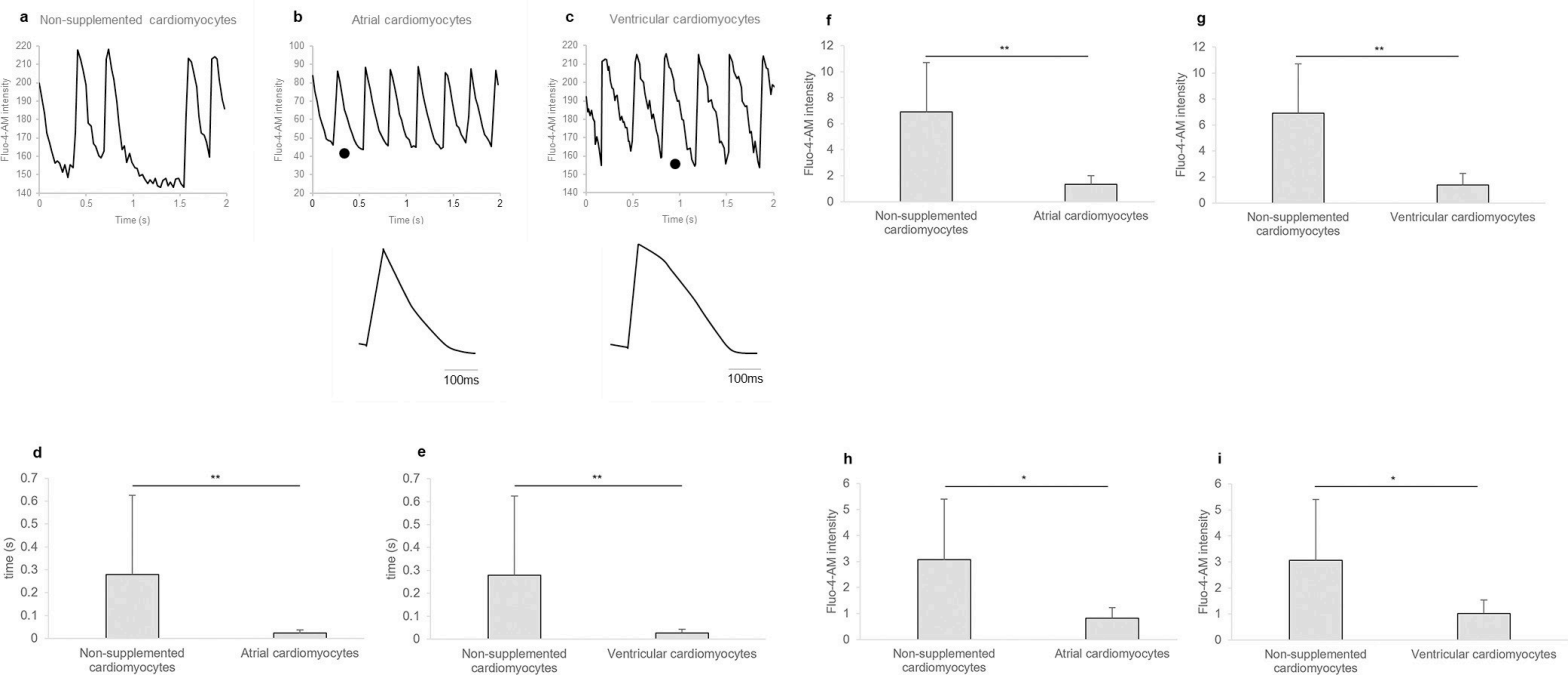
Regular contraction of atrial and ventricular cardiomyocytes differentiated from rat HAP stem cells. (a-c) Intracellular Ca^2+^ (Fluo-4-AM) imaging of spontaneously beating cardiomyocytes differentiated from HAP stem cells. Lower, calcium transients shown at an expanded timescale taken from the region indicated (•) in the upper. (a) Irregular contractions of non-supplemented cardiomyocytes. (b, c) Representative waveforms of atrial and ventricular cardiomyocytes. (d, e) Dispersion in calcium transient duration of cardiomyocytes differentiated from HAP stem cells. n = 17 for number of standard deviation for non-supplemented cardiomyocytes beats (n = 51 for number of non-supplemented cardiomyocytes beats), n = 25 for number of standard deviation for atrial cardiomyocytes beats (n = 75 for number of atrial cardiomyocytes beats), n = 27 for number of standard deviation for ventricular cardiomyocytes beats (n = 81 for number of ventricular cardiomyocytes beats). Data are presented as mean ± SD. ** P < 0.001, two-sided Mann-Whitney U test. (f, g) Dispersion in calcium transient amplitude of cardiomyocytes differentiated from HAP stem cells. n = 17 for number of standard deviation for non-supplemented cardiomyocytes beats (n = 51 for number of non-supplemented cardiomyocytes beats), n = 25 for number of standard deviation for atrial cardiomyocytes beats (n = 75 for number of atrial cardiomyocytes beats), n = 27 for number of standard deviation for ventricular cardiomyocytes beats (n = 81 for number of ventricular cardiomyocytes beats). Data are presented as mean ± SD. ** P < 0.001, two-sided Welch’s t-test. (h, i) Dispersion in resting calcium concentration of cardiomyocytes differentiated from HAP stem cells. n = 17 for number of standard deviation for non-supplemented cardiomyocytes beats (n = 51 for number of non-supplemented cardiomyocytes beats), n = 27 for number of standard deviation for atrial cardiomyocytes beats (n = 81 for number of atrial cardiomyocytes beats), n = 27 for number of standard deviation for ventricular cardiomyocytes beats (n = 81 for number of ventricular cardiomyocytes beats). Data are presented as mean ± SD. * P < 0.005, two-sided Welch’s t-test.

All cardiomyocytes differentiated from HAP stem cells showed spontaneous beating (S2 Video). Fluorescence microscopy of the cardiomyocytes loaded with the calcium-sensitive dye Fluo-4-AM showed that the intracellular Ca^2+^ concentration varied in response to beating of the cardiomyocytes (Fig 4A). Characteristic waveforms were observed in atrial and ventricular cardiomyocytes differentiated from HAP stem cells. Atrial cardiomyocytes differentiated from HAP stem cells lacked a clear plateau and showed a higher frequency of spontaneous activity than ventricular cardiomyocytes (Fig 4B). Ventricular cardiomyocytes differentiated from HAP stem cells demonstrated rapid calcium transient rising phase and a plateau phase (Fig 4C). Atrial or ventricular cardiomyocytes differentiated from HAP stem cells had significantly less variance in calcium transient duration (Fig 4D and 4E) (*p* < 0.001, *p* < 0.001 respectively), in calcium transient amplitude (Fig 4F and 4G) (*p* < 0.001, *p* < 0.001 respectively) and in resting calcium concentration (Fig 4G and 4I) (*p* = 0.002, *p* = 0.001 respectively) than non-supplemented cardiomyocytes differentiated from HAP stem cells.

## Discussion

The differentiation of atrial or ventricular cardiomyocytes is critical for regenerative medicine for heart disease. Atrial or ventricular cardiomyocytes have been induced from embryonic stem cells (ESC) and induced pluripotent stem cells (IPSC). Yan et al. previously reported that the addition of CSA to ESC [18] and Fujiwara et al. to IPSC [19] improved the differentiation efficiency of cardiomyocytes and cardiomyocyte progenitor cells. In the present study, we selectively differentiated rat HAP stem cells into atrial cardiomyocytes in culture supplemented with the combination of isoproterenol, activin A, bone morphogenetic protein 4 (BMP4), basic fibroblast growth factor (bFGF), and cyclosporine A (CSA), suggesting that rat HAP stem cells may have a propensity to become atrial cardiomyocytes. Lee et al. induced differentiation of ventricular cardiomyocytes from IPSC by adding inhibitor of Wnt production-2 (IWP2), and vascular endothelial growth factor (VEGF) [14]. In the present study, we differentiated ventricular cardiomyocytes from rat HAP stem cells in culture supplemented with inhibitor of Wnt production-4 (IWP4) and VEGF. Rat HAP stem cells can be differentiated into atrial or ventricular cardiomyocytes in culture controlled by specific supplementations without genetic manipulation which are mature as demonstrated in this study.

A previous study showed that in the adult mouse and human heart, *MLC2a* is strongly expressed in the atria but less (or absent) in the ventricles, in contrast to *MLC2v*, which is expressed in the ventricles but is weakly (or absent) in the atria [20]. Immunostaining demonstrated that atrial cardiomyocytes differentiated from rat HAP stem cells are MLC2a positive and MLC2v negative, while ventricular cardiomyocytes differentiated from rat HAP stem cells are MLC2v positive and MLC2a negative. Both atrial and ventricular cardiomyocytes had regular sarcomere structures similar to ESC- and IPSC-derived cardiomyocytes [21].

Ventricular cardiomyocytes also showed T-tubule-like structures, indicating that these were mature cardiomyocytes. ESC- and IPSC-derived atrial or ventricular cardiomyocytes are spindle-shaped or star-shaped. Compared to these cells, atrial or ventricular cardiomyocytes differentiated from rat HAP stem cells formed long, polygonal myocardial fibers and beat as a functional syncytium as shown in S1 Video, which had a structure more similar to adult cardiomyocytes.

Intervening plates were identified in ventricular cardiomyocytes differentiated from rat HAP stem cells by positive β-catenin staining, but not visible by transmission electron microscopy. Although cardiomyocytes differentiated from rat HAP stem cells had fewer intervening plates than cardiomyocytes in vivo or differentiated from IPSC and ESC [19, 22], cardiomyocytes differentiated from rat HAP stem cells were in close contact with each other and contracted as a syncytium.

Atrial or ventricular cardiomyocytes differentiated from rat HAP stem cells showed regular waveforms by Ca^2+^ imaging, which indicated that there was a mechanism for smooth communication between adjacent cardiomyocytes. Furthermore, atrial or ventricular cardiomyocytes differentiated from rat HAP stem cells had significantly stable contractions, resting calcium concentration and calcium transient amplitude, compared to the non-supplemented cardiomyocytes differentiated from rat HAP stem cells, which showed irregular contractions and unstable resting calcium concentration and calcium transient amplitude. Atrial or ventricular cardiomyocytes differentiated from rat HAP stem cells also exhibited characteristic waveforms of atrial and ventricular cardiomyocytes [15, 16, 21], respectively, which confirmed that rat HAP stem cells can differentiate into both atrial and ventricular cardiomyocytes.

Although HAP stem cells are located in the ectoderm, specifically in the hair follicles, our study has already shown that HAP stem cells are differentiated into vascular endothelial cells [23] and cardiomyocytes, which belong to the mesoderm by culturing only in 10% FBS-DMEM. The present study demonstrates that HAP stem cells, which are adult stem cells, can be differentiated into atrial or ventricular cardiomyocytes without genetic manipulation demonstrating future clinical potential. Our study has also demonstrated that human HAP stem cells also have pluripotency and form SSEA3 -, SSEA4 -, Oct3/4 -, NANOG -, and nestin -positive spheres [24]. These results indicate a high potential for differentiation of atrial and ventricular cardiomyocytes from human HAP stem cells.

HAP stem cells are promising for clinical application because they can be autologously transplanted without the ethical and tumorigenic issues that ESC and IPSC have. These results are expected to promote research on cardiotoxicity screening, drug discovery, and regenerative medicine by cardiomyocyte subtypes.

## Supporting information

S1 Video SiR-actin live-cell imaging of beating cardiomyocytes differentiated from rat HAP stem cells. (MP4)

S2 Video Beating cardiomyocytes differentiated from rat HAP stem cells. (MP4)

S1 Data Source data for all graphs in main text. (XLSX)
